# Estimating the Demographic Parameters of *Tuta absoluta* (Lepidoptera: Gelechiidae) Using Temperature-Dependent Development Models and Their Validation under Fluctuating Temperature

**DOI:** 10.3390/biology11020181

**Published:** 2022-01-24

**Authors:** Samira Abuelgasim Mohamed, Abdelmutalab G. A. Azrag, Francis Obala, Shepard Ndlela

**Affiliations:** 1 International Centre of Insect Physiology and Ecology (*icipe*), P.O. Box 30772, Nairobi 00100, Kenya; sfaris@icipe.org (S.A.M.); fobala@icipe.org (F.O.); or shepardndlela@gmail.com (S.N.); 2 Department of Crop Protection, Faculty of Agricultural Sciences, University of Gezira, P.O. Box 20, Wad Medani 21111, Sudan

**Keywords:** tomato, climate warming, ILCYM, phenology model, life cycle, developmental biology

## Abstract

**Simple Summary:**

*Tuta absoluta* is an invasive insect pest that has spread widely and established itself in many countries since its first detection in Spain in 2006. The devastating moth originates in South America and attacks tomato and other solanaceous vegetables, leading to huge losses in yield and potential income particularly for small-scale farmers who often lack the resources and knowledge to manage the pest. In most cases, farmers have resorted to the indiscriminate application of broad-spectrum synthetic pesticides, which in most cases are not registered and are often used at high doses. This has resulted in the pest developing resistance to most major classes of pesticides. In addition, the non-selective use of toxic pesticides has resulted in negative effects on the health of users, consumers, and non-target organisms such as pollinators and natural enemies of insect pests. Various tactics aimed at controlling *T. absoluta* have been developed and are at different stages of adoption by farmers. To ensure that they are effective, sustainable, and friendly to both users and the environment, there is a need for a comprehensive understanding of the pest’s biology and ecology. To this effect, the present study developed models to predict intricate details of the pest’s development, survival, and reproduction using data generated in laboratory studies. Among other important findings, the study reports that temperatures between 20–25 °C are ideal for the development, survival, reproduction, and increase in the population of *T. absoluta.* These findings are vital in developing strategies in managing the pest, especially in light of global climate change.

**Abstract:**

The tomato leafminer, *Tuta absoluta* (Meyrick) (Lepidoptera: Gelechiidae) is an invasive pest that devastates the production of tomatoes and other solanaceous vegetables. Since its trans-Atlantic invasion in 2006, *T. absoluta* has spread and established in many countries across the Afro-Eurasian Supercontinent, causing huge yield losses. This study aimed to determine the relationship between temperature and the life history traits of *T. absoluta* and provide the thermal thresholds for development using life cycle modelling. Linear and non-linear models were fitted to life table data collected at five constant temperatures of 15, 20, 25, 30, and 35 °C, with Relative Humidity 70 ± 5% and photoperiod 12L:12D. Another experiment was conducted at fluctuating temperatures to validate the laboratory results. *Tuta absoluta* completed its life cycle at temperatures between 15 and 35 °C. The development time ranged between 4.0–11 days, 6.3–16.0 days, and 5.4–20.7 days for egg, larva, and pupa, respectively. The lowest thermal threshold was estimated at 8.10, 7.83, and 11.62 °C, respectively for egg, larva, and pupa. While the optimum temperature for *T. absoluta* immature stages survival and female fecundity were predicted at a temperature range of 21–23 °C. The intrinsic rate of increase (*r_m_*), gross reproductive (*GRR*), and net reproductive (*Ro*) rates were significantly higher at temperatures between 20–25 °C. The model validation outcome showed similarities between observed and simulated values for development time, mortality rate, and life table parameters, attesting to the quality of the phenology model. Our results will help in predicting the effect of climate warming on the distribution and population dynamics of *T. absoluta*. Furthermore, the results could be used to develop management strategies adapted to different agroecological zones.

## 1. Introduction

The tomato leaf miner, *Tuta absoluta* (Meyrick) (Lepidoptera: Gelechiidae) is one of the most destructive insect pests of tomato and other solanaceous crops worldwide [1,2]. The larvae feed on the vegetative parts of tomato plants and fruits, causing yield loss ranging between 80–100% [3]. Since its trans-Atlantic invasion in 2006, *T. absoluta* has spread and established itself in many countries across the Afro-Eurasian Supercontinent [4,5,6]. In Africa, *T. absoluta* was detected in Morocco in 2008 and has since then rapidly spread across the continent, causing devastating losses, especially for resource-poor small-scale farmers who could not suppress the pest’s populations [7,8]. The high dispersal ability [9] coupled with its wide thermal range (8 °C and 35 °C) for development [10,11], and high reproduction rate [12] have contributed to the successful invasion and subsequent establishment of *T. absoluta* in its new invaded range.

Due to the economic impact of *T. absoluta* in the horticultural industry, several attempts have been made to tackle this menace. These include mass trapping using sex pheromones [13,14], mating disruption [15], and agronomic practices, especially the use of field sanitation [3,16]. Biological control, using the egg parasitoids *Trichogramma* spp., was also used to control *T. absoluta* in North Africa [2]. Similarly, some natural enemies were identified as biological control agents against *T. absoluta* in the invaded areas, however, they failed to keep the pest infestation below the economic damage level. For example, two larval parasitoids, *Bracon nigricans* Szépligeti (Braconidae) and *Dolichogenidea appellator* (Telenga) (Braconidae) were reported to have attacked *T. absoluta* in Sudan, however parasitism was too low to effectively manage the pest [17]. A recent study by Aigbedion-Atalor et al. [18] revealed the promising performance of the exotic larval parasitoid, *Dolichogenidea gelechiidivoris* (Marsh) (Hymenoptera: Braconidae) as a potential classical biological control agent of *T. absoluta* in Africa. Nevertheless, all these management options are currently not effective enough to control *T. absoluta*. Chemical control using synthetic insecticides remains the main option for the control of this pest [7]. However, insecticides are not a sustainable management option due to their adverse effects on non-target organisms and the development of pest resistance [19,20], as well as adverse effects on both human and environmental health [21]. Thus, there is a need for the development and adoption of alternative management measures to control *T. absoluta* in the horticultural industry. In this regard, the development of novel control strategies of *T. absoluta* should incorporate its life-history traits and ecology to effectively manage the pest based on the specific agro-ecological zones.

Considering that insects are poikilothermous, temperature is the most important factor affecting their biology and ecology. Insects’ development, survival, reproduction, population growth, distribution, and population dynamics are temperature-dependent [22]. Through phenotypic plasticity, insects are able to change their physiological activities and behavior in response to changes in the surrounding environmental conditions [23,24]. This plasticity is driven by various physiological factors including enzymes and hormonal regulations that result in local or systemic reactions [24,25]. These reactions can be modelled by regressing the values for the phenotypic trait against the environmental factor, a process known as reaction norms [25]. The reaction norm has been widely used to visualize the responses of life-history traits of different insects against environmental conditions including temperature [22,26,27]. This is an important tool for understanding the phenotypic variation in insects and predicting their distribution and abundance in light of global warming [28,29].

The success of invasion and the establishment of alien pests such as *T. absoluta* in new environmental conditions is attributed to various factors, among which are the phenotypic plasticity traits that play a vital role in the fitness and survival of the pest [30]. Several studies have attempted to evaluate the effect of constant temperatures on the development, survival, and reproduction of *T. absoluta* [10,11,31,32,33,34,35]. These studies focused only on predicting the reaction norm for the developmental rate using linear degree-day and non-linear models. However, they did not consider the simulation of the development time, mortality, and fecundity of *T. absoluta* that allows for the estimation of the population growth rate. In addition, the developed non-linear models for *T. absoluta* immature stage were not validated under a fluctuating temperature to be used in the prediction of distribution and the potential risk of the pest. Therefore, this study aims to investigate the effect of temperature on the development, survival, and reproduction of the Kenyan population of *T. absoluta* using linear and non-linear models and to simulate the life table parameters.

## 2. Materials and Methods

### 2.1. Tuta Absoluta Rearing

The insect colony used in this study was initiated by collecting *T. absoluta* infested tomato plants (Cal J and Anna F1 varieties) from Kirinyaga (S 00°36′24.8″ E 037°22′30.0″) and Nakuru (S 00°49′28.4″ E 036°32′42.9″) counties, Kenya. The collected tomato samples were incubated in insect rearing Perspex cages (50 cm × 40 cm × 60 cm) and maintained in the laboratory under ambient conditions (25 ± 2 °C, 70 ± 5% RH, 12L:12D regime) at the International Centre of Insect Physiology and Ecology (*icipe*), Nairobi, Kenya. Clean uninfested tomato leaves, purposefully grown in insect-proof screenhouses under standard agronomic practices without the application of any fertilizers or insecticides, were added, when necessary, to the incubation cages until moth eclosion. Upon adult emergence, the moths were aspirated into clean Perspex cages (50 cm × 40 cm × 60 cm) holding three potted plants (≥6-weeks old). Natural honey (100%) droplets were streaked on the top underside of the cages using a fine camel-hair brush and moistened cotton wool was occasionally provided for nourishment. The moths were allowed to oviposit on the tomato plants for 48 h, and thereafter the infested plants were replaced with clean uninfested potted tomato plants for oviposition. The *T. absoluta* infested plants (confirmed by the presence of eggs on the leaves) were removed from the cage and placed on a working bench under ambient conditions (25 ± 2 °C, 70 ± 5% RH, 12L:12D regime) until egg hatching. After hatching, the infested plants were maintained under the same ambient laboratory conditions until the second larval instar. Leaves with third instar larvae were cut and transferred into a clean Perspex cage lined with a paper towel at the bottom to prevent dumping and to provide a surface for pupation. Upon eclosion, moths were aspirated into clean Perspex cages, where they were provided with fresh tomato for regeneration of the colony using the same method as described above. This rearing strategy allowed us to maximize the insect rearing space, where several infested plants were placed on the working bench and leaves with second instar larvae were cut and placed in incubation cages, which could not hold as many potted plants as the infested leaves. The moths were reared for three generations before they were used in the experiment.

### 2.2. Effects of Temperature on the Developmental Time of Immature Stages

The effect of temperature on the developmental time of *T. absoluta* immature stages was studied in incubators set at five constant temperatures of 15, 20, 25, 30, and 35 °C as a cohort life table. Tomato plant leaves with 100-newly laid eggs (≤24 h old) were cut and placed in a transparent plastic box (2-litres capacity), hereinafter referred to as an experimental unit, lined with a paper towel at the bottom. The eggs were carefully counted, and any excess was gently removed from the leaves using a soft camel-hair brush. The experimental units were individually introduced into incubators set at constant temperatures of 15, 20, 25, 30 and 35 °C (±0.5 °C), and humidity at 70 ± 5% and 12L:12D photoperiod regimes. The choice of this temperature range was based on our pilot studies, the temperature range for tomato production [36], and the fact that the thermal tolerance of most insect species in the tropical region ranges between 13 and 35 °C [37,38,39]. Each incubator had five replicates (experimental units), bringing the total number of eggs used at each temperature to 500 eggs. Eggs were monitored daily at each temperature to record the incubation period. The eggs that failed to hatch were kept for an additional 10 days before they were dissected under a stereomicroscope (Leica WILD M3Z) using dissecting pins, to confirm the presence or absence of the embryo. The dead eggs at each constant temperature were confirmed by the presence of the dead embryo inside the shell and they were recorded.

To assess the developmental times of *T. absoluta* larvae, 10 newly emerged larvae (≤24 h old) from the eggs that were placed at the constant temperatures were collected and placed into the experimental unit and provided with three tomato leaves of an equal number of leaflets and placed in the incubators set at constant temperatures described above. Five replicates of the same procedure were undertaken for the five constant temperatures, bringing the total number of larvae used at each temperature to 50. Fresh tomato leaves were provided every two days and the developmental time of larvae was monitored daily until pupation.

The developmental time from pupation to adult eclosion was also assessed. Ten newly formed *Tuta absoluta* pupae from the eggs that were placed at the constant temperatures were collected and placed into a vial (Soda Glass Poly Stopper; 75 × 25 mm) capped with the stopper. Then five vials (one vial being a replicate) holding the pupae were placed at each incubator set at one of the experimental temperatures described above. Moth eclosion was monitored and recorded daily. Uneclosed pupae were dissected under the stereoscope to confirm mortality and the dead pupae were counted and recorded.

### 2.3. Effect of Temperature on Adult Longevity

Newly formed pupae were collected from the colony and individually sexed according to the method described by Genc [40]. This was necessitated by the fact that it was not possible to obtain enough adults from the initial eggs that had hatched at some particular temperatures i.e., 35 °C, hence we used newly formed pupae from the colony maintained at (25 ± 2 °C). Both the female and male pupae (at least 70 pupae) were placed in specimen tubes and maintained under the experimental temperature regimes. Upon adult emergence, 20 moths (10 males and 10 females) were transferred into clean Perspex cages (20 × 20 × 20 cm), provided with natural honey droplets carefully streaked on the top underside of the cage using a fine camel-hair brush and water in form of moistened cotton wool. The setup was replicated five times for both females and males for each temperature. Fresh honey and moistened cotton wool were introduced every three days. Adult longevity was monitored and recorded daily until all the moths died.

### 2.4. Oviposition by T. absoluta Females

Pupae were sexed as described in the above section and placed at each constant temperature until eclosion. Newly emerged moths were paired (1♀:1♂) and placed into Perspex oviposition cages (20 × 20 × 20 cm), which were then introduced into the incubators set at the experimental temperatures described above (Section 2.2). Food was provided in the form of fine droplets of honey placed on the top underside of the cages, while water was provided in form of moistened cotton wool placed at the bottom of the cage. Potted tomato plants (about 12 cm in height) were introduced into the oviposition cages. The plants were removed daily from the cages and replaced with new ones. The number of eggs laid by each female during the 24 h was counted and recorded. The females were maintained until all of them died. However, in the case of a male death occurring before the female, it was replaced by another male (≤3 days of the age range of female). At each temperature, the experiment was replicated 30 times.

### 2.5. Development, Survival, and Reproduction under Fluctuating Temperature

A similar experimental procedure as the one described for constant temperatures was conducted under a fluctuating temperature in semi-natural conditions at the *icipe* (S 01°22′05.1″; E 036°89′56.3″, and altitude 1619 m above sea level). This experiment was conducted to validate the developed phenology model with data collected under constant temperatures. Three hundred newly laid eggs (≤24 h old) were collected from the same colony described above and placed in five Perspex cages each containing 60 eggs. The cages with eggs were placed in an open field with a fluctuating temperature and semi-natural conditions and remained under these same conditions until the end of the experiment. After egg hatching, the larvae, pupae, and adults from these experimental units were used to evaluate the effect of fluctuating temperature on the development of *T. absoluta*. The developmental time and mortality of larva and pupa, as well as adult longevity and female fecundity, were monitored using the same methods described above in Section 2.2, Section 2.3 and Section 2.4 for constant temperature experiments. A HOBO data logger was installed in the Perspex cages to record daily minimum and maximum temperatures at one-hour intervals. The daily minimum and maximum temperature during experimentation ranged from 11.6 to 18.6 °C and from 20.6 to 38.6 °C, respectively.

### 2.6. Modelling Program

Insect Life Cycle Modelling (ILCYM, version 3.0) program [41] was used to develop a temperature-dependent phenology model for *T. absoluta* using life table data obtained from different constant temperatures. This software contains three modules: (i) model builder module that consists of various non-linear models that are fitted to characterize temperature-dependent development, survival, and reproduction of the insect pest; (ii) validation and simulation module, which is used to validate the phenology model with life table data collected at fluctuating temperature; and (iii) population analysis and mapping module, which is used to predict the impact of climate change on the distribution and abundance of the pest, based on the developed and validated phenology model. Notably, the validation and simulation module uses deterministic and stochastic simulations to estimate the life table parameters that determine the population growth rate of the pest, based on the developed and validated phenology model. In this study, we used model builder and validation and simulation modules to develop and validate the phenology and simulated the life table parameters. We fitted several non-linear models for each development parameter and thereafter we selected the best-fit one based on the coefficient of determination (R^2^) and the Akaike’s Information Criterion (AIC) [41]. The Least Square Design (LSD) test was used at α = 0.05 for each fitted parameter in ILCYM to determine the probability thresholds of the fitted model.

#### 2.6.1. Development Time and Its Variation

The developmental times and adult longevity (in days) of *T. absoluta* at each constant temperature were ln-transformed. Then, cumulative frequency distributions were plotted against normalized development times and fitted to cumulative density functions. The Complementary log-log (CLL) function was the best for egg, pupa, female, and male, while Logit function was the best for the larva stage. The mathematical equations for these two functions are given in Table 1.

#### 2.6.2. Development Rate of Immature Stages

The developmental rate was calculated at each constant temperature for each immature stage by inversing the developmental times (in days). The linear model, r(T)=a+bT, was fitted to describe the linearity of the development rate, where r(T) is the development rate at temperature T; a and b are the intercept and the common slope, respectively. Using this model, the minimum temperature for the development $\left(T_{min}\right)$ was estimated for each immature stage as $T_{min}=-a/b,\;\;$ while the degree-days (DD) thermal constant was calculated as DD=1/b. The linear model failed to estimate the upper thermal threshold for the development. Hence non-linear models were used to evaluate the relationship between temperature and the development rate at high temperatures. We fitted fifty-nine non-linear models for each immature stage development rate, and then the best-fitted model was selected based on R^2^ and AIC, combined with the biological sense of the pest. The best-fitted model was Sharpe and DeMichele function 6 [42] for egg and larva, and Briere function 1 [43] for the pupa stage. The mathematical equations for these models are given in Table 1.

#### 2.6.3. Mortality Rate

The mortality of *T*. *absoluta* immature stages was calculated at each constant temperature and then fitted to forty-nine non-linear models. The best-fitted model that described temperature-dependent mortalities of all *T.* *absoluta* immature stages was Wang function 1 [44]. The mathematical equation of Wang function 1 model is given in Table 1.

#### 2.6.4. Female Fecundity and Adult Senescence

To assess age-specific fecundity, the cumulative fecundity of the females was plotted against the females’ age. Then, the cumulative proportion of eggs produced at each age was fitted to Exponential modified function 3, for which the mathematical expression of the model presented is in Table 1. In addition, the number of eggs laid per female at each constant temperature was fitted to Taylor function 1 (Table 1) to describe temperature-dependent fecundity. Adult senescence (female and male), which is referred to as adult survival, was calculated for each constant temperature by inversing the adult longevity and then fitted to an exponential simple function to assess the effect of temperature on males and females’ senescence rate, for which the mathematical equation is given in Table 1.

#### 2.6.5. Life Table Parameters

All developed temperature-dependent models for *T.* *absoluta* development, mortality, female fecundity, and adult senescence were compiled in one phenology model. Then, using the ‘stochastic simulation’ module in ILCYM, the following life table parameters that determine the population growth rate were estimated at tested temperatures: (1) the gross reproductive rate (*GRR*); (2) the net reproductive rate (*R_o_*); (3) the intrinsic rate of natural increase (*r_m_*); (4) the mean generation time (*Tc*); (5) the doubling time (*D_t_*); and (6) the finite rate of increase (λ) [41]. The simulations started with 100 individuals at the egg stage and were replicated 5 times for each tested temperature.

#### 2.6.6. Model Validation

To validate the phenology model, daily minimum and maximum temperatures were recorded from the same experimental site, where a *T. absoluta* life table was collected at fluctuating temperatures. Then, development and the life table parameters of *T. absoluta* were simulated using the developed phenology model and recorded daily fluctuating temperatures. We generated a life table with 100 individuals at the egg stage from the developed phenology model to simulate these parameters. The output of this process (simulated values from the phenology model) was compared with observed values obtained from the life table data conducted at fluctuating temperatures. The differences between the observed and simulated values were used to test the validity of the developed phenology model. The similarities between the observed and simulated values indicate that the developed phenology model is valid and could be used for further studies such as the prediction of the distribution and abundance of the pest under climate warming [41].

### 2.7. Statistical Analysis

The effect of constant temperatures on the developmental time of *T*. *absoluta* immature stages (in days) and adult longevity (in days) was analyzed using GLM with Poisson distribution performed using the package MASS [45] in R version 3.6.3 [46]. The estimated life table parameters were checked for normality using Shapiro–Wilk tests, then, were subjected to one-way ANOVA. If a significant difference was found, SNK test at α = 0.05 was used to separate the means. A t-test was used to compare the longevity of the males and females at each tested temperature.

## 3. Results

### 3.1. Development Time and Adult Longevity

*Tutaabsoluta* development occurred at a constant temperature between 15 and 35 °C. The observed incubation period decreased significantly with an increase in temperature (χ^2^ = 863.04, *df* = 2175, *p* < 0.0001) (Table 2). Similarly, temperature had profound effect on larval developmental time (χ^2^ = 70.434, *df* = 154, *p* < 0.0001) being shortest at 35 °C (6.3 days) and longest (16.1 days) at 15 °C. Also, the observed developmental time of pupal stage significantly varied across the temperatures (χ^2^ = 125.71, *df* = 342, *p* < 0.0001) (Table 2), again being shortest (5.4 day) and longest (20.7 day) at 35 and 15 °C, respectively. Likewise, longevity of both *T. absoluta* sexes differed with temperature (χ^2^ = 279.81, *df* = 241, *p* < 0.0001 and χ^2^ = 282.93, *df* = 247, *p* < 0.0001, for males and females, respectively) (Table 2). The longevity of the males and females was similar at 20, 25 and 35 °C, however it was significantly different at 15 °C (*t* = 6.8735, *df* = 51.302, *p* < 0.0001) and 30 °C (*t* = −4.9637, *df* = 65.211, *p* <0.0001).

The cumulative frequency distribution of the development times for egg and pupa, as well as female and male longevity, was well fitted to a CLL model (R^2^ = 0.90–98; AIC = 294–1766) (Supplementary Table S1; Supplementary Figure S1). However, the developmental time-frequency for the larval stage was well described by Logit function (R^2^ = 0.92; AIC = 222) (Supplementary Table S1; Supplementary Figure S1). The simulated development times by the models for all immature stages and adult longevity were similar to the observed developmental times obtained from the experimental data (Table 2), indicating the quality of fitted models. 

### 3.2. Development Rate

**Figure 1:**
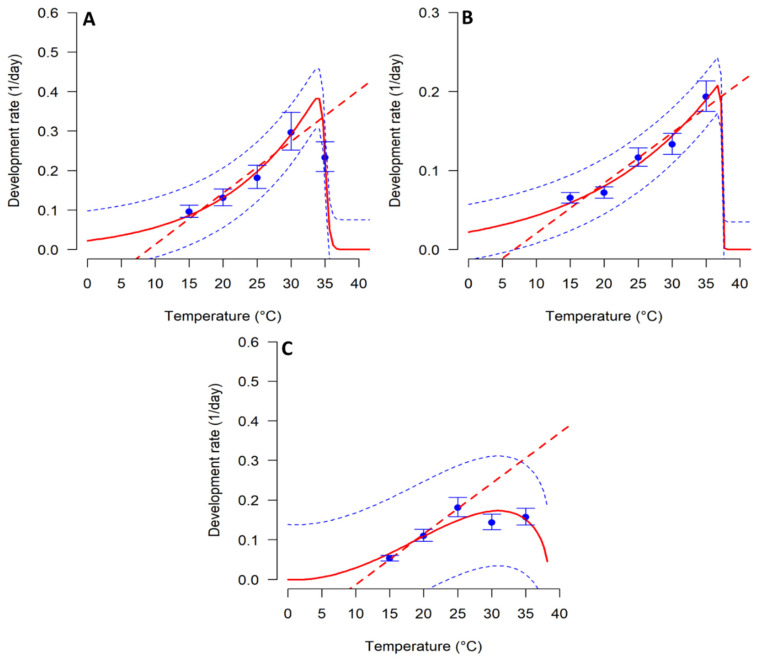
Temperature-dependent development rates of *Tuta absoluta* immature stages fitted to Sharpe and DeMichele function 6 for (**A**) egg, (**B**) larva, and Briere function 1 for (**C**) pupa. The blue points represent the experimental data with bars representing the standard deviation. Fitted models are the red broken lines for linear models and solid lines for the non-linear models. The broken blue lines above and below represent the upper and lower 95% confidence interval.

Temperature significantly affected the developmental rate of *T.* *absoluta* immature stages (Table 3; Figure 1). The linear model was well fitted to the developmental rate of all immature stages (R^2^ between 0.96 and 0.99). The lowest temperature threshold for the development of the immature stages was estimated from the linear model at 8.10, 7.83, and 11.62 °C, for eggs, larvae, and pupae, respectively. The degree-days thermal constant, which refers to the amount of energy needed for each stage to complete its development to the next phase was estimated to be 100, 166.67, and 73.53 degree-days for egg, larva, and pupa, respectively. Among the 59 non-linear models that were fitted to temperature-dependent development rate, Sharpe and DeMichele function 6 was the best for egg (R^2^ = 0.93; AIC = −11.846; *p* < 0.0001) and larval (R^2^ = 0.87; AIC = −4.204; *p* < 0.0001) stages (Table 3; Figure 1A,B). On the other hand, Briere function 1 was the best fitted for the pupal stage (R^2^ = 0.79; AIC = −16.779; *p* < 0.0001) (Table 3; Figure 1C).

### 3.3. Mortality Rate

**Figure 2:**
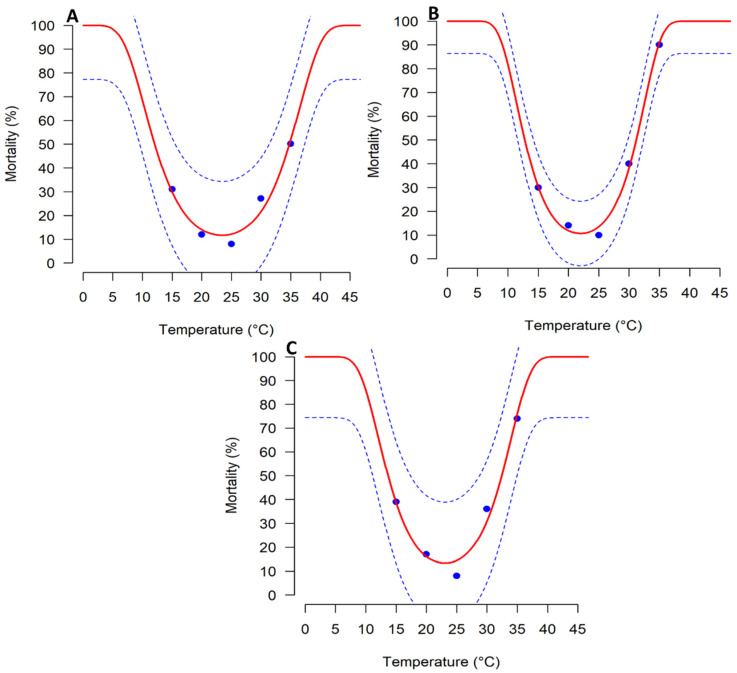
Temperature-dependent mortality rates of *Tuta absoluta* immature stages fitted to Wang function 1: (**A**) egg; (**B**) larva; (**C**) pupa. The blue points represent the experimental data. The solid red lines represent non-linear models and broken blue lines above and below represent the upper and lower 95% confidence interval.

The mortality rate of *T*. *absoluta* life stages was significantly affected by the temperature (Table 4; Figure 2). The highest and the lowest mortality rates were reported at 35 and 25 °C, respectively for all life stages (Figure 2). The relationship between *T.* *absoluta* immature stages mortality and the temperature was well determined by Wang function 1 (R^2^ = 0.95 and 0.99 and AIC = −11.81 and −16.96) (Table 4; Figure 2). This function estimated the optimal temperature $(T_{opt}$), in which the lowest mortality rate of immature stages occurs at 23.42, 21.99, and 23.06 °C for egg, larva, and pupa, respectively (Table 4; Figure 2).

### 3.4. Female Fecundity and Adult Senescence

**Figure 3:**
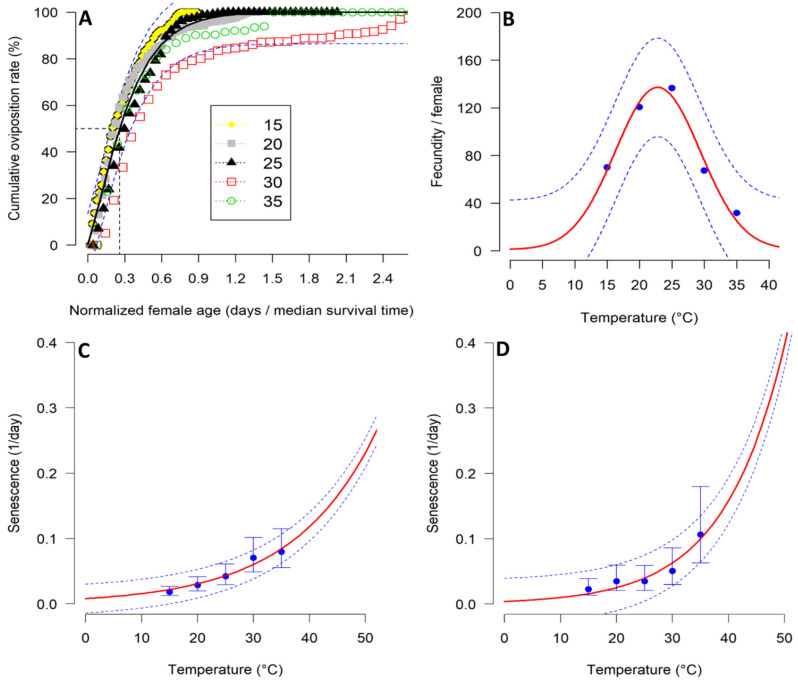
Model fitting to determine the relationship between temperature and *Tuta absoluta* female fecundity, and adult senescence: (**A**) cumulative fecundity fitted to Exponential modified function 3; (**B**) mean fecundity per female fitted to Taylor function 1; (**C**) female senescence rates fitted to Exponential simple function; and (**D**) male senescence rates fitted to Exponential simple function. The blue points represent the experimental data with bars representing the standard deviation. The solid red lines represent non-linear models. The broken blue lines above and below represent the upper and lower 95% confidence intervals.

The cumulative fecundity of *T.* *absoluta* females in relation to the age of the female was well assessed by the Exponential modified function 3 (R^2^ = 0.93, AIC = −601.49) (Table 5; Figure 3A). Temperature had a profound influence on *T.* *absoluta* fecundity (Table 5). Overall, the highest fecundity was obtained at 25 °C, with an average of 136.6 eggs per female (Figure 3B). The temperature-dependent fecundity was well fitted to Taylor function 1 (R^2^ = 0.97; AIC = 40.24). The function predicted that the optimal temperature for *T.* *absoluta* oviposition ranged between 22 and 23 °C (Figure 2B). Temperature also had a significant effect on the senescence of both sexes of *T.* *absoluta* (Table 5). The temperature-dependent senescence for both males and females were well described by Exponential simple function (female: R^2^ = 0.94; AIC = −32.08) (Figure 3C), and male (male: R^2^ = 0.91; AIC = −27.39). 

### 3.5. Life Table Parameters

**Figure 4:**
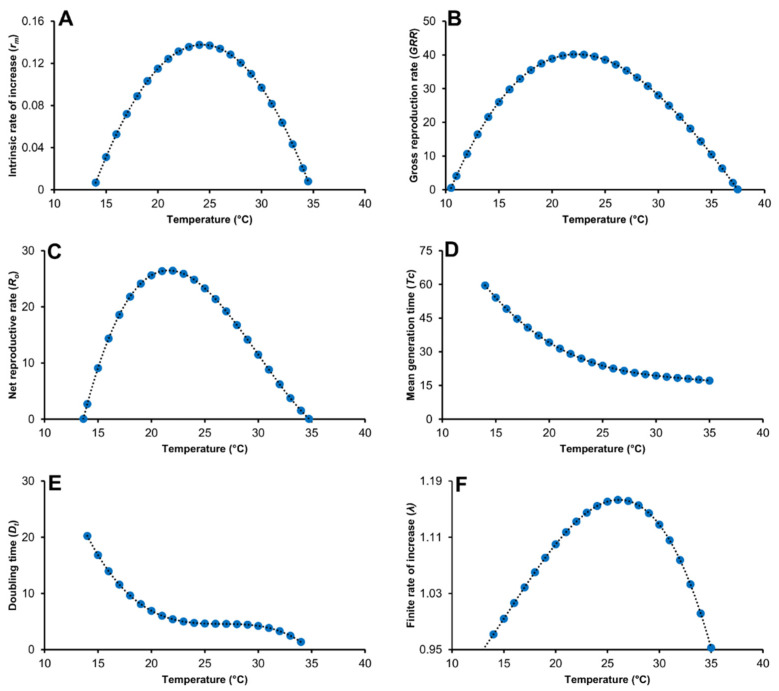
Simulated life table parameters of *Tuta absoluta* under constant temperatures using 1 °C interval, with (**A**) intrinsic rate of natural increase, (**B**) gross reproductive rate, (**C**) net reproduction rate, (**D**) mean generation time (in days), (**E**) doubling time (in days) and (**F**) Finite rate of increase.

Temperature significantly influenced the life table parameters of *T.* *absoluta*. The intrinsic rate of increase (*r_m_*) was significantly higher at constant temperatures of 20 and 25 °C, compared to the other tested temperatures (Table 6). The highest gross reproductive rate (*GRR*) was reported at 20° C with 38.88 daughters per female, followed by 25 °C, while the lowest was reported at 35 °C (Table 6). The net reproductive rate (*R_o_*) significantly increased at 20 and 25 °C, and thereafter it decreased at 30 °C (Table 6). Temperature also had a significant effect on the mean generation time (*Tc*), which decreased with an increase in temperature. The same trend was observed for the doubling time (*Dt*), which represents the time required for the *T.* *absoluta* population to double itself. The finite rate of increase (*λ*) ranged from 0.94 at 35° C to 1.14 at 25 °C (Table 6). The temperature range, between 20 and 27 °C, was the most favorable for *T.* *absoluta* development, survival, and reproduction, in which high population growth occurs, with a short time between generations and quick population doubling (Figure 4).

### 3.6. Model Validation

**Figure 5:**
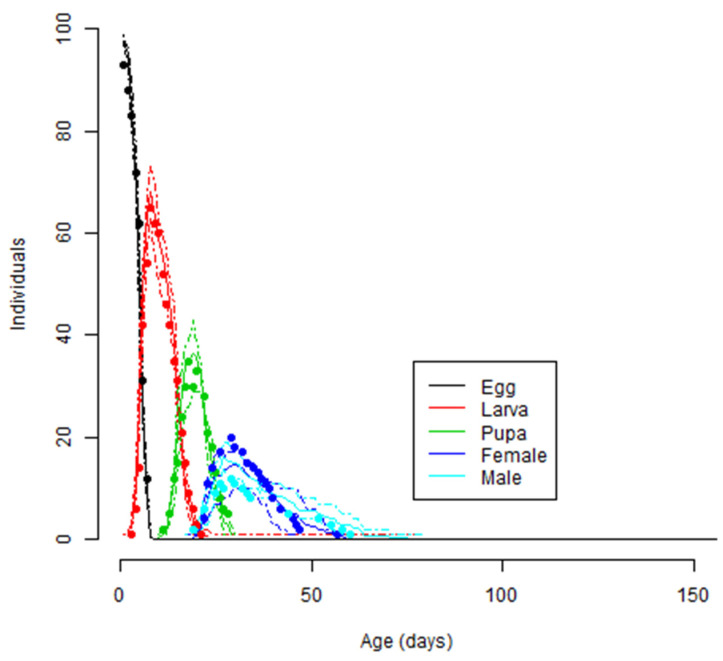
Observed and simulated *Tuta* *absoluta* life stage frequencies for testing the validity of temperature-dependent development models fitted to the development, survival, and reproduction. The lines represent simulated values for each life stage obtained from the phenology model, while the dots represent observed values for each life stage obtained from the experiments conducted at fluctuating temperatures.

The observed developmental time and mortality of *T. absoluta* immature stages, as well as the life table parameters obtained from the experiment conducted at fluctuating temperatures and those predicted from the fitted phenology models, are shown in Figure 5 and Table 7. The results showed consistency between the observed and simulated values for *T.* *absoluta* development, mortality, and population growth parameters (Table 7). The simulated development times were 4.99, 9.95, and 8.06 days, compared to the observed development times of 5.25, 10.0, and 8.03 days for eggs, larva, and pupa, respectively (Table 7). In addition, life table parameters also showed huge similarities between the observed and simulated values (Table 7). The frequencies of the observed values for *T. absoluta* developmental stages were well aligned with those of simulated values, attesting to the quality of the fitted models (Figure 5).

## 4. Discussion

Temperature is known to be the most important environmental factor that affects the development, survival, reproduction, distribution, abundance, and population dynamics of insects [39,47]. In this study, we simulate the complete life history traits of *T.* *absoluta* using the phenology modelling approach, unlike other studies that only simulated the development time and rate at constant temperatures [10,11,31,32,33], or estimated the life table parameters to determine the population growth rate [34,35]. Our results showed that *T. absoluta* completed its life cycle at temperatures between 15 °C to 35 °C. However, the response of *T. absoluta* life stages to temperature differed, which indicates that the thermal requirements vary between the life stages. The findings of this study provide greater insight into the temperature-dependent development of *T*. *absoluta* since its invasion of the African continent. In light of global warming, an increase in temperature might induce a shift in the synchrony between the insect pests and their host plants or natural enemies, thus influencing interspecific interactions [47,48,49]. Also, temperature rise is expected to change the distribution, abundance, population dynamics, and range expansion of many insect species with potentially severe consequences for biological invasion [47,50]. In this regard, estimation of the thermal requirements and population growth parameters of the pest is very relevant for developing pest management strategies and the prediction of species distributions under climate warming. Therefore, the results obtained in this study could be used to predict the effect of global warming on the invasion risk and distribution of *T.* *absoluta* based on temperature using ILCYM [47,51]. 

Our results for *T*. *absoluta* egg incubation period (4.3 to 11.6 days) agree with those reported by Bentancourt et al. [52] and Krechemer and Foerster [10]. However, the incubation period we obtained at 15 °C was longer than that reported by de Campos et al. [53] who recorded 9.3 days at the same temperature. In contrast, the development times of larvae at all tested temperatures were much shorter than those reported by Bentancourt et al. [52], who recorded development times of 11.2 and 29.7 days at 5 and 30 °C, respectively. Contrary to our study, the larval stage did not complete its development at 35 °C as in the study by Bentancourt et al. [52]. Similarly, de Campos et al. [53] reported 32.1 days at 15 °C and 10.1 days at 33 °C, which was longer than our findings. This variation in larval development time could be attributed to the differences in the experimental conditions, where de Campos et al. [53] used relative humidity of 60 ± 5% and photoperiod 16:08 h L:D, while we used 70 ± 5% relative humidity and 12:12 h L:D photoperiod. In addition, the study by de Campos et al. [53] and Bentancourt et al. [52] used tomato seedlings grown with fertilizer application, compared to our seedlings that were free from fertilizer application. For example, it has been demonstrated that an increase in nitrogen content in tomato leaves significantly decreases the larval developmental time of the leaf miner *Liriomyza trifolii* [54]. Therefore, the difference in food quality provided for *T. absoluta* larvae could be responsible for the observed differences. The development time for the pupa stage was similar to those reported by Krechemer and Foerster [10] and de Campos et al. [53].

Temperature-driven models are important tools for predicting the thermal response (reaction norms) of insect development under different climatic regimes. Although insects do not develop at a constant temperature in nature, temperature-driven models provide adequate ecological information such as the thermal thresholds that can be used to predict the pest distribution and population dynamics. For example, the temperature thresholds for pest development obtained from such studies are important for simulating their development under field conditions [55]. Since control measures against insect pests are more effective in the susceptible life stage, temperature-driven models could predict the phenology of the insect species, identify the vulnerable stage, and also could be used to estimate the timing between control measures and the presence of vulnerable stages of the pest in the field for effective management strategy [50,55]. In the present study, we used linear and non-linear models to assess the impact of temperature on life history traits of *T.* *absoluta*. The development rate increased linearly in the temperature range between 15 to 30 °C for egg and larval stages, and between 15 to 25 °C for the pupal stage. This linear trend was similar to those reported by Krechemer and Foerster [10]; Özgökçe et al. [33] and de Campos et al. [53] for the same stages of the pest. The lowest temperature thresholds for egg and larval development were relatively close to those reported by Krechemer and Foerster [10] who obtained 9.6 °C and 6.7 °C for egg and larva, respectively. However, our findings contrast with those of de Campos et al. [53], who predicted the minimum temperature threshold for *T.* *absoluta* population from France at 5.7, 5.1, and 5.3 °C for egg, larva, and pupa, respectively. These variations in minimum temperature thresholds may be explained by the fact that *T*. *absoluta* used in these experiments are from different populations adapted to different climate regions, thus a difference in thermal response is expected. 

In the current study, the non-linear Sharpe and DeMichele function [42] gave the best fit for the egg and larva, while Briere function 1 [43] was the best-fitted model for the pupal stage. Our study confirms the findings of Rossini et al. [56] who suggested the Sharpe and DeMichele model as the best function for simulating the development rate of *T.* *absoluta*. The biological importance of this function is that it can forecast the development rate of insects based on the enthalpy of enzyme activation [42]. Furthermore, the Sharpe and DeMichele function can be fitted in a variety of ways (four, five, six, and seven parameters), making it adaptable to various temperature levels [41,57]. On the other hand, the Briere function is one of the non-linear models that have been widely used to predict insect development rates. Unlike the DeMichele function, the Briere function has the ability to estimate all the three temperature thresholds for insect development (minimum, optimum, and maximum temperature thresholds) [43]. In our study, the non-linear functions estimated the maximum temperature thresholds to be approximately 36, 38, and 38 °C, for egg and larva and pupa, respectively. These thresholds were higher than those reported by Krechemer and Foerster [10], who predicted the maximum temperature thresholds to be at 35.1, 34.3, and 36 °C, for egg, larva, and pupa, respectively. Similarly, our estimates were different from all models’ outputs fitted by Marchioro et al. [11]. These differences in thermal requirements could be due to the different *T.* *absoluta* populations as well as the different functions used in the prediction. For example, Krechemer and Foerster [10] used the Lactin function, while Marchioro et al. [11] used the Analytics, Briere, Janisch, Lactin, Logan, and Polynomial functions to estimate the thermal thresholds. In general, these variations suggest that *T.* *absoluta* has high thermal plasticity to different climatic conditions. Indeed, a study by Tarusikirwa et al. [30] revealed that the larval stage of *T.* *absoluta* has high plasticity to the minimum and maximum temperatures. This thermal plasticity is likely one of the contributing factors to the successful invasion in many African countries with different climatic conditions in a short time.

In the present study, the temperature-dependent mortality showed that the mortality rate of *T.* *absoluta* immature stages was higher at extreme temperatures. This is attributed to the fact that the physiological, biochemical, and metabolic reactions of insects are highly influenced by the temperature of their surrounding environment [48,58]. The Wang function 1 [44] predicted the optimum temperature for *T.* *absoluta* immature stages survival between 21 to 23 °C, in which the mortality rate was between 10–13%. This model was also adopted for predicting the mortality of many insects in tropical regions such as the coffee berry borer *Hypothenemus hampei* (Ferrari) (Coleoptera: Curculionidae, Scolytinae) and the African coffee white stem borer *Monochamus leuconotus* (Pascoe) (Coleoptera: Cerambycidae) [57,59], as well as the mealybug *Phenacoccus solenopsis* Tinsley (Hemiptera: Pseudococcidae) [60].

In this study, we obtained average fecundity of 71.1, 120.7, 136.6, 67.4, and 31.8 eggs per female at constant temperatures of 15, 20, 25, 30, and 35 °C, respectively. The fecundity of *T.* *absoluta* was high at temperatures ranging between 20 and 25 °C and sharply decreased at 35 °C. This decline at high temperatures could be due to the short longevity of the female as well to the effect of the high temperature on female’s maturity resulting in low fecundity [61]. Since the adults we used for fecundity assessment were obtained from pupae formed in the colony, the results we obtained should be interpreted with caution as the rearing of eggs and larvae at ambient conditions (25 ± 2 °C) might have influenced the female oviposition. A study by Mahdi and Doumandji [31] recorded average fecundity of 122 at 15 °C, which was much higher than our findings at the same temperature. Similarly, the maximum oviposition we obtained (136 eggs per female) was twice lower than that reported by Uchoa-Fernandes et al. [12] who obtained a maximum fecundity of 260 eggs per female. However, our results were comparable to those reported by Krechemer and Foerster [10] for the temperature range of 15 to 25 °C, with the number of eggs per female being 76.4, 134.8, 149 at 15, 20, 25, respectively. The fitted Taylor function 1 estimated that *T.* *absoluta* females could lay eggs at a temperature range between 6 and 38 °C, with 22.8 being the optimum temperature for oviposition. The temperature range we reported in the current study is considered fairly wide for a tropical pest, like *T*. *absoluta*, a trait that may have aided in its adaptability to various agroecological zones across tropical and temperate regions. The thermal range for fecundity we obtained (6 and 38 °C) was different from the report of de Campos et al. [53], who predicted a thermal window between 5.4 and 35.7 °C using polynomial functions. The differences between the two studies could be explained by the two different models fitted to fecundity, which may result in the estimation of different thermal thresholds. Nevertheless, the results of our model could have been affected by over or underestimated fecundity due to the rearing of early stages (eggs and larvae) of the females at ambient conditions (25 ± 2 °C).

The effect of temperature on bionomics of *T. absoluta* has strongly manifested on the life table parameters, such as the intrinsic rate of natural increase (*r_m_*). We reported a negative *r_m_* (−0.065) at 35 °C, which indicates that *T. absoluta* population growth is adversely impacted at this temperature. The net reproductive rate (*R_o_*) we obtained deviated from those reported by de Campos et al. [53], whereby the authors recorded *R_o_* between 25.2 and 36.5 at a temperature range of 15–25 °C. This could be attributed to the lower mortality rate of immature stages in the study by de Campos et al. [53], compared to ours. However, our results were similar to those reported by Martins et al. [34]. In the present study, the mean generation time ranged between 19 and 54 days, which indicates that *T. absoluta* can complete between 6 to 15 generations per year in constant temperatures ranging between 15 and 30 °C.

Since insects do not develop under a constant temperature in their natural habitat, the developed temperature-dependent phenology for *T.* *absoluta* under different constant temperatures experiments was validated using daily fluctuating temperature records. The life table experiment conducted under fluctuating temperatures showed that *T.* *absoluta* has a strong amount of potential to grow its population despite the daily temperature variations, which ranged between 11.6 and 38.6 °C, with a mean annual of 23.1 °C. In this fluctuating temperature range, the *r_m_* was 0.103, with *R_o_* of 16 daughters per female suggesting a high growth population rate of the pest. In tropical regions, the mean annual temperature ranges between 25 and 30 °C [62,63], a conducive environment for *T. absoluta* population to rapidly grow and thrive, and this could explain the rapid invasion of the pest in the tropical region. Indeed, *T.* *absoluta* can complete 13 consecutive generations per year under this temperature range depending on the availability of host plants. The comparable outcome of the observed and simulated values we obtained for the development time, mortality, and demographic parameters (such as the intrinsic rate of natural increase) proved that the developed phenology models for *T.* *absoluta* are a best fit for simulating the developmental parameters of the pest. Therefore, these models can be used to predict the impact of global warming in the future distribution and abundance of *T.* *absoluta*.

## 5. Conclusions

In conclusion, our study used a phenology modelling approach to simulate the complete life cycle of *T.* *absoluta* under both constant and fluctuating temperatures, the results of which showed that temperature has a significant effect on the biological parameters of *T.* *absoluta*. The findings of the present study will help in understanding the role of temperature on the development, survival, and population growth of the pest. In addition, our models could be used to predict the effect of climate warming on the distribution and population dynamics of *T.* *absoluta*. 

## Figures and Tables

**Table 1:** Temperature-dependent models that were used to determine the effect of temperature on *Tuta absoluta* immature development time, development rate, mortality rate, male and female longevity, and senescence, and female oviposition. All the mathematical equations for the fitted functions are given by Tonnang et al. [41].

Life History Traits	Model Name	Model Equation	Equation No	Life Stage Fitted to
Development time	CLL distribution function	$f\left(x\right)=1-\exp\left(-\exp\left(a_{i}+b\ln x\right)\right)$	(1)	Egg, Pupa, Female, and Male
	Logit distribution function	$f\left(x\right)=1/(1+\exp\left(-\left(a_{i}+b\ln x\right))\right)$	(2)	Larva
Development rate	Sharpe and DeMichele 6	$r\left(T\right)=\frac{P\times \frac{T}{T_{o}}\times \;\exp\left[\frac{\Delta H_{A}}{R}\left(\frac{1}{T_{o}}-\frac{1}{T}\;\right)\right]}{1+\;\exp\left[\frac{\Delta H_{H}}{R}\;\left(\frac{1}{T_{H}}-\frac{1}{T}\right)\right]}$	(3)	Egg and Larva
	Briere 1	$r\left(T\right)=aT\left(T-T_{o}\right)(\sqrt{T_{max}-T}$)	(4)	Pupa
Mortality rate	Wang 1	$m\;\left(T\right)=1-\frac{1}{\exp\;\left((1+\exp(-\frac{T-T_{opt}}{B}))\times (1+\exp(-\frac{T_{opt}-T}{B}))\times H\right)}$	(5)	Egg, Larva, and Pupa
Cumulative fecundity	Exponential modified 3	$f\left(x\right)=1-\exp\left(-a\times T^{b}\right)$	(6)	Female
Fecundity/female	Taylor 1	$f\left(x\right)=1-rm\times \exp(-\frac{1}{2}(-\frac{\left(T-T_{opt}\right)}{T_{roh}}{)}^{2}\;)$	(7)	Female
Senescence	Exponential simple	$s\left(T\right)=b_{1}\times \exp(b_{2}\times T)$	(8)	Female and Male

**Table 2:** Mean (±SE) observed developmental times calculated from the experimental data and simulated developmental times (in days) estimated from the model for immature stages of *Tuta absoluta* reared at different constant temperatures.

T (°C)	Egg		Larva		Pupa		Female Longevity		Male Longevity	
	Observed	Simulated	Observed	Simulated	Observed	Simulated	Observed	Simulated	Observed	Simulated
15	11.60 ± 0.17 a	10.44 ± 0.22 a	16.06 ± 0.30 a	17.22 ± 0.18 a	20.68 ± 0.38 a	19.71 ± 0.25 a	56.12 ± 4.09 aA	54.13 ± 2.06 a	48.53 ± 4.49 aB	45.58 ± 2.00 a
20	8.04 ± 0.07 b	7.85 ± 0.16 b	14.60 ± 0.31 a	14.06 ± 0.19 b	8.94 ± 0.29 b	9.09 ± 0.17 b	36.46 ± 2.07 bA	34.91 ± 1.52 b	32.45 ± 1.91 bA	29.58 ± 1.71 b
25	5.91 ± 0.05 c	5.50 ± 0.12 c	9.29 ± 0.37 b	8.63 ± 0.15 c	5.41 ± 0.10 d	5.53 ± 0.09 d	25.00 ± 1.45 cA	24.67 ± 1.04 c	28.44 ± 2.19 bcA	28.77 ± 1.67 b
30	4.04 ± 0.05 d	3.93 ± 0.08 d	7.88 ± 0.34 b	8.38 ± 0.14 c	6.54 ± 0.08 c	6.98 ± 0.12 c	14.90 ± 0.82 dB	14.19 ± 0.53 d	22.02 ± 1.99 cA	21.76 ± 1.27 c
35	4.33 ± 0.05 d	4.30 ± 0.09 d	6.25 ± 1.03 d	6.79 ± 0.11 d	6.81 ± 0.18 bc	6.38 ± 0.09 c	11.29 ± 0.74 eA	12.56 ± 0.57 e	11.16 ± 0.30 dA	9.41 ± 0.42 d

Means in each column followed by the same letter are not significantly different (SNK test, α = 0.05). The observed developmental time refers to the experimental data, while simulated development time refers to those simulated by the model. For male and female longevity, means in each row followed by the same capital letter are not significantly different.

**Table 3:** Estimated parameters of Sharpe and DeMichele 6 and Briere 1 models fitted to determine the relationship between temperature and the development rate of immature stages *Tuta absoluta* reared at different constant temperatures.

Life Stage	Function	Model Parameters		*F*	*df.*	*p*	R^2^	AIC
Egg	Sharpe and DeMichele 6	P	0.1827 ± 0.0092	16.73	4, 10	<0.0001	0.87	−4.204
		*To*	297.1867 ± 0.0001					
		*HA*	13,493.05 ± 0.000					
		$H_{H}$	627,028.7 ± 0.000					
		*TH*	308.209 ± 0.06574					
Larva	Sharpe and DeMichele 6	P	0.1125 ± 0.0036	31.81	4, 10	<0.0001	0.93	−11.846
		*To*	298.8563 ± 0.00002					
		*HA*	9683.435 ± 0.000					
		$H_{H}$	2,555,621 ± 0.000					
		*TH*	310.4786 ± 0.000					
Pupa	Briere 1	a	0.0001 ± 0.000	3.72	2, 2	0.212	0.79	−16.779
		$T_{o}$	2.1892 ± 0.000					
		$T_{max}$	38.4074 ± 0.000					

**Table 4:** Estimated parameters for Wang function 1 fitted to determine the relationship between temperature and the mortality rate of *Tuta absoluta* immature stages.

Life Stage	$T_{opt}$	B	H	F	*df.*	*p*	R^2^	AIC
Egg	23.42 ± 0.63	3.77 ± 0.53	0.03 ± 0.01	19.36	2, 2	0.0491	0.95	−11.81
Larva	21.99 ± 0.29	2.93 ± 0.19	0.03 ± 0.00	206.26	2, 2	0.0048	0.99	−16.96
Pupa	23.06 ± 0.51	3.29 ± 0.38	0.04 ± 0.01	35.60	2, 2	0.0273	0.97	−10.61

**Table 5:** Estimated parameters of Exponential modified function 3, Taylor function 1 and Exponential simple function 3 fitted to determine the relationship between temperature and the female fecundity and adult senescence *Tuta absoluta* reared at different constant temperatures.

Parameter	a	b	rm	$T_{opt}$	$T_{roh}$	$b_{1}$	$b_{2}$	*F*	*df.*	*p*	R^2^	AIC
Cumulative fecundity	3.73 ± 0.18	1.24 ± 0.04						3252.85	1, 238	<0.0001	0.93	−601.49
Fecundity/female			−136.17 ± 8.0	22.81 ± 0.45	6.56 ± 0.51			38.47	2, 2	0.0253	0.97	40.24
Female senescence						0.01 ± 0.00	0.07 ± 0.01	55.04	1, 3	0.0014	0.94	−32.08
Male senescence						0.01 ± 0.00	0.09 ± 0.02	32.45	1, 3	0.0114	0.91	−27.39

**Table 6:** Simulated life table parameters of *Tuta absoluta* population reared at five constant temperatures. The intrinsic rate of increase (*r_m_*), gross reproduction rate (*GRR*), net reproduction rate (*R*_0_), mean generation time (*Tc* in days), doubling time (*Dt*) in days, and finite rate of increase (*λ*).

Temperature (°C)	*r_m_*	*GRR*	*R*_o_	*Tc*	*Dt*	Λ
15	0.041 ± 0.001 c	25.85 ± 1.16 b	9.33 ± 0.79 b	54.21 ± 0.49 a	16.99 ± 0.57 a	1.04 ± 0.001 c
20	0.126 ± 0.003 a	38.88 ± 2.38 a	24.99 ± 1.95 a	33.66 ± 0.25 b	5.49 ± 0.13 c	1.13± 0.003 a
25	0.129 ± 0.004 a	37.82 ± 1.99 a	24.62 ± 1.69 a	24.69 ± 0.53 c	5.37 ± 0.16 c	1.14 ± 0.004 a
30	0.095 ± 0.003 b	27.51 ± 1.44 b	11.12 ± 0.63 b	19.00 ± 0.11 d	7.29 ± 0.23 b	1.10 ± 0.003 b
35	−0.065 ± 0.013 d	9.22 ± 1.94 c	0.36 ± 0.07 c	17.55 ± 0.30 e	-	0.94 ± 0.012 d
Statistics						
*F*	143.49	42.59	73.25	2448.09	99.33	166.95
*df.*	4, 20	4, 20	4, 20	4, 20	4, 20	4, 20
*p*	<0.0001	<0.0001	<0.0001	<0.0001	<0.0001	<0.0001

Means in each column followed by the same letter are not significantly different (SNK test, α = 0.05).

**Table 7:** Comparison between the observed values obtained from the experiment at fluctuating and predicted values obtained from the fitted temperature-based development models of *Tuta absoluta*.

Parameters		Simulated Values	Observed Values	*p*-Value
Development time (in days)	Egg	4.99 ± 0.18	5.25	0.323
	Larva	9.95 ± 0.39	10.00	0.539
	Pupa	8.06 ± 0.53	8.03	0.771
Mortality (%)	Egg	23.8 ± 0.72	21.5	0.146
	Larva	34.1 ± 0.84	37.0	0.114
	Pupa	65.2 ± 0.81	71.7	0.003
Life table parameters	Intrinsic rate of increase (*r_m_*)	0.092 ± 0.008	0.103	0.063
	Gross reproduction rate (*GRR*)	43.334 ± 15.717	54.201	0.006
	Net reproduction rate (*R*_o_)	12.08 ± 3.303	16.200	0.051
	Mean generation time (*Tc*)	26.934 ± 1.655	27.077	0.6741
	Doubling time (*Dt*)	7.553 ± 0.636	6.739	0.083
	Finite rate of increase (*λ*)	1.096 ± 0.009	1.108	0.140

## Data Availability

The data presented in this study are available within the article.

## Supplementary Materials

The following are available online at https://www.mdpi.com/article/10.3390/biology11020181/s1, Table S1: Estimated parameters (intercept and common slope) of complementary log-log (CLL) and Logit functions fitted to the cumulated frequency distributions of development times of *Tuta absoluta* immature stages and adult longevity reared at five constant temperatures. Figure S1: Cumulative frequency distribution of the developmental times of *Tuta absoluta* fitted to (A) egg, (B) larva, (C) pupa, (D) female, and (E) male. Curves are fitted models and the bars represent 95% confidence intervals for median development times estimated from the models.
