# Supplementary material for: Estimating the Demographic Parameters of Tuta absoluta (Lepidoptera: Gelechiidae) Using Temperature-Dependent Development Models and Their Validation under Fluctuating Temperature
Source: Biology (Basel). 2022 Jan 24;11(2):181. doi: 10.3390/biology11020181 (PMC8869599; doi:10.3390/biology11020181)
Supplement: Supplementary file 1 [file biology-11-00181-s001.zip › biology-1509129-supplementary.pdf]

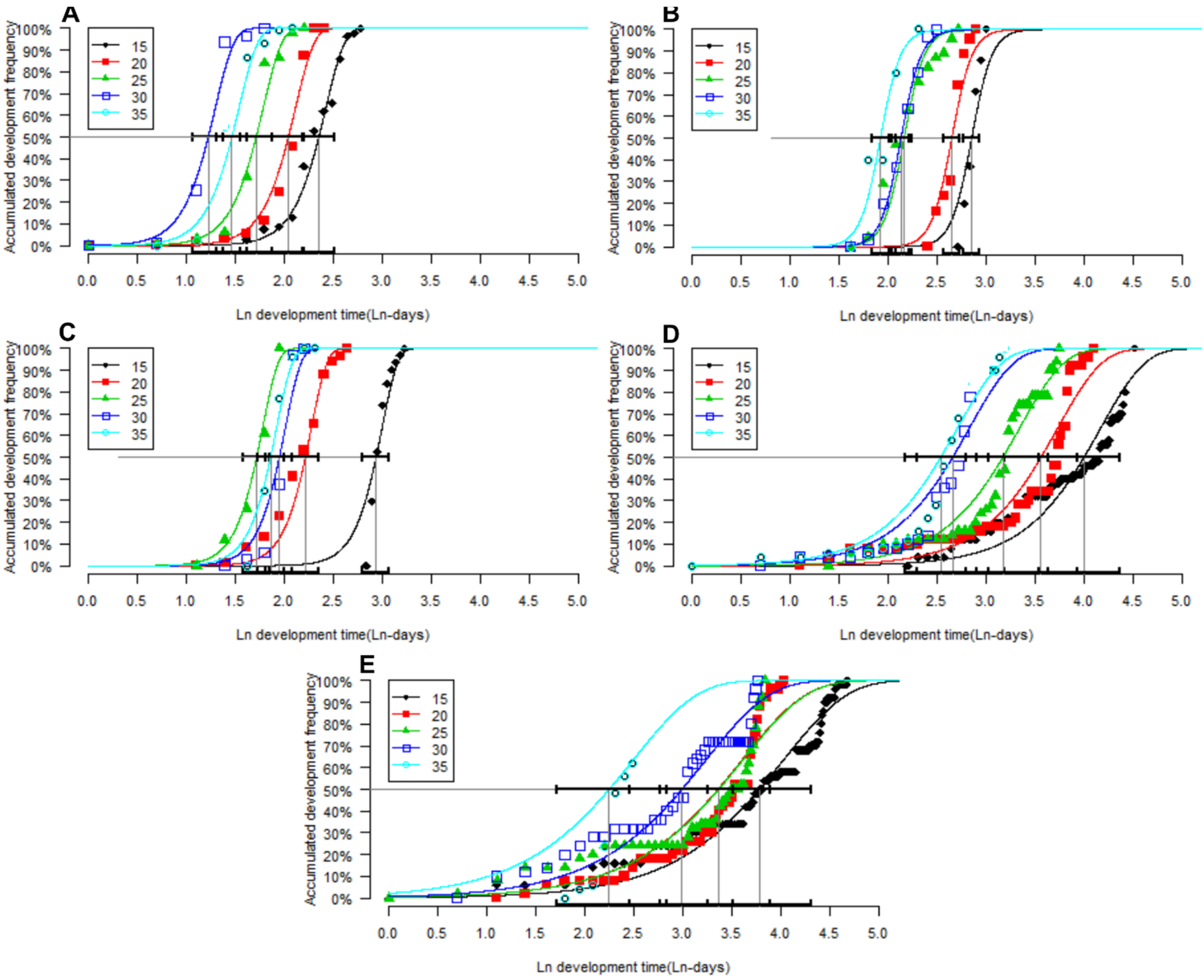

Supplementary Figure S1. Cumulative frequency distribution of the developmental times of *Tuta absoluta* fitted to A) egg, B) larva, C) pupa, D) female and E) male. Curves are fitted models and the bars represent 95% confidence intervals for median development times estimated from the models.

Supplementary Table S1. Estimated parameters (intercept and common slope) of complementary log-log (CLL) and Logit functions fitted to cumulative frequency distributions of development times of *Tuta absoluta* immature stages and adult longevity reared at 5 constant temperatures.

Life Stage	y-Intercept (*a*)					Slope (*b*)	R^2	AIC
	15°C	20°C	25°C	30°C	35°C			
Egg1	-11.39 ± 0.15	-9.44 ± 0.13	-8.03 ± 0.11	-6.09 ± 0.09	-6.58 ± 0.09	4.529 ± 0.06	0.98	912.88
Larva2	-22.55 ± 1.18	-21.76 ± 1.14	-17.77 ± 0.94	-16.6 ± 0.89	-13.59 ± 0.91	8.26 ± 0.43	0.92	222.18
Pupa1	-18.36 ± 0.78	-13.93 ± 0.53	-10.88 ± 0.42	-12.30 ± 0.47	-11.75 ± 0.47	6.14 ± 0.23	0.94	297.15
Female1	-9.13 ± 0.18	-8.16 ± 0.16	-7.31 ± 0.15	-6.19 ± 0.13	-5.92 ± 0.13	2.19 ± 0.04	0.91	1451.04
Male1	-6.13 ± 0.12	-5.49 ± 0.11	-5.4 ± 0.11	-4.92 ± 0.10	-3.79 ± 0.09	1.53 ± 0.023	0.90	1766.44
